# Sexual selection leads to positive allometry but not sexual dimorphism in the expression of horn shape in the blue wildebeest, *Connochaetes taurinus*

**DOI:** 10.1186/s12862-022-02060-3

**Published:** 2022-09-11

**Authors:** Chloé Gerstenhaber, Andrew Knapp

**Affiliations:** 1 University College, London, UK; 2 The Natural History Museum, London, UK; 3 Queen Mary University of London, London, UK

**Keywords:** Sexual selection, Evolution, Geometric morphometrics, Mammalia

## Abstract

Sexual selection is thought to be an important driver of adaptation, speciation and extinction. Empirically testing these predictions across macroevolutionary timescales first requires an understanding of the morphology of secondary sexual traits in extant taxa. We used three-dimensional geometric morphometrics to analyse a large sample of the skull of the blue wildebeest, *Connochaetes taurinus*, in which horns are found in both sexes but only used in intrasexual competition in males. We show that the horns fit several predictions of secondary sexual traits; overall skull shape is significantly correlated with size (R^2^ = 0.38, *p* = 0.001), and the sexually selected horns show drastically higher growth rates and variation than any other skull element, supporting previous findings. We also find that despite showing significant sexual dimorphism in shape and size (R^2^ = 0.21, *p* = 0.001), allometric growth trajectories of sexes are identical (R^2^ = 0.01, *p* = 0.635) and dimorphism is not readily detectable without prior knowledge of sex, and is not possible when shape is corrected for size. Our results show that even with strong sexual selection operating in only one sex, the expression of secondary sexual traits may show characteristic and indistinguishable patterns of growth and variance in both sexes.

**Supplementary Information:**

The online version contains supplementary material available at 10.1186/s12862-022-02060-3.

## Background

Sexual selection arises from competition for fertilisation opportunities and is responsible for the evolution of diverse secondary sexual traits in the animal kingdom, including exaggerated morphologies, behaviours and strategies [1–3]. Sexual selection is expected to have a powerful effect on evolution, speciation and extinction [4–7], and although much theoretical and laboratory work has been done to test these predictions, exploring these effects over macroevolutionary timescales is more challenging. Incorporating fossil data into these studies is a possible solution but requires identification of sexually selected traits in fossil taxa based on morphology alone; our incomplete knowledge of these taxa makes this difficult in practice [8]. The biology of extant organisms is generally much better understood and can be used to explore the effects of sexual selection on morphology.

Secondary sexual traits are known to display characteristic patterns of growth and variation that distinguish them from functionally constrained naturally selected traits [9]. For example, traits which act as visual signals often show positive static allometry, being proportionally larger in sexually mature individuals [10]. This is revealed as a slope of greater than 1 when trait size is regressed against body size [11]. This phenomenon is widely observed in secondary sexual traits across the animal kingdom and is thought to arise by being an ‘honest signal’, because growing and maintaining proportionally larger traits is more efficient for larger individuals [12–14]. Studies of secondary sexual traits in extant taxa tend to focus on the competing sex, which may impede their application to extinct taxa, where sex is often unknowable [15–17]. Moreover, striking sexual dimorphism seen in many secondary sexual traits has led some researchers to suggest that such traits can only be identified by the presence of sexual dimorphism [17, 18]. Nonetheless, sexual dimorphism, particularly in fossil taxa, can be difficult to detect and may be hampered by incomplete knowledge of taxa [15]. Furthermore, examples exist of extant taxa in which a secondary sexual trait is expressed in both sexes of a species but may only perform a sexually selected role in one, further complicating the identification of these traits [19–22].

Differing roles of secondary sexual traits between sexes may lead to selection for positive allometry in these traits being relaxed in one sex, leading to dimorphism in growth and variation between sexes [20, 23, 24]. Conversely, some secondary sexual traits may also perform a range of functions connected with social selection, which may operate in a similar way to sexual selection in both sexes [19, 21, 26]. Furthermore, there is a tendency in studies of sexually selected traits to focus just on these traits, neglecting phenotypic complexity elsewhere and potentially biasing results towards such ‘exaggerated’ traits [27]. Traditional one-dimensional linear measurements of trait size are often employed in studies of sexually selected traits, but univariate data cannot account for more complex changes in shape with size or in differences in growth elsewhere in the organism [28]. The exaggerated growth and high variation typical of secondary sexual traits [11] suggest that they are largely free of the narrower functional constraints of other, naturally selected traits, and consequently they are likely to be weakly integrated with the rest of the organism [29]. Phenotypic modularity, the tendency of sets of traits to form integrated ‘modules’ which covary more strongly than with other integrated sets of traits, can further help to determine interactions between morphological traits by assessing their integration across an organism [30]. Sexually selected traits, such as horns, should form distinct modules because relaxed integration with the rest of the skull would allow them to respond to selection with some degree of independence [29], and can explain the extraordinary diversity of these traits across the animal kingdom [3]. Determining integration of traits across an organism may therefore be an important indicator of function, especially when attempting to identify sexually selected traits. Modern geometric morphometric (GM) techniques allow the analysis of shape across a number of associated traits and can therefore capture more of the phenotypic complexity of the skull [28].

The ruminant family Bovidae comprises 143 extant species. The males of all species bear sexually selected horns growing from the frontal bones which are constructed of a bony core, the os cornu, covered by a horn sheath formed of keratinised epidermis [31]. Bovid horn shape appears to be correlated with male fighting style in intrasexual competition [32]. Although female bovids do not physically compete for mates and are therefore not expected to be subject to sexual selection in the same way as males, female horns are found in roughly half of all extant bovid species [19, 33]. It is thought that female bovid horns are maintained by either natural selection (e.g. predator defence), social selection (e.g. territoriality, male mimicry), or by genetic linkage to male horns [19]. The biology of many bovid taxa are well understood and specimens are readily available in museum collections, making them an ideal study group for investigating intraspecific variation of secondary sexual traits. The blue wildebeest, *Connochaetes taurinus,* is a medium-sized bovid in the tribe Alcelaphini [34]. Sexes resemble each other, with females being generally smaller than males and with less robust horns [33–35]. *C. taurinus* is divided into five subspecies spread across East and Southern Africa, often living in very large populations which can in turn lead to intense competition between males for mates. Male *C. taurinus* hold small territories which they defend with ritualised aggressive behaviour, and horns are used in these aggressive displays and in physical competition with other males, with larger males generally being dominant [35]. The prominent role of horns in these contests suggest that, although obvious weapons, they may also have an important display function and thus may show the predicted positive allometry and increased variance of a secondary sexual display trait [10, 11, 36]. There is some disagreement over the role of horns in female *C. taurinus,* with predator defence and male mimicry being leading hypotheses, and they are not known to be used in physical competition between females [35]. Although the horns of *C. taurinus* are known to show positive allometry in length in males [11], it is not known if this relationship is seen in females, nor how shape is related to size either in horns or in the skull in general.

Using three-dimensional (3D) geometric morphometrics, we analyse a large sample of *C. taurinus* skulls of both sexes and a range of sizes to test the following predictions:

1. Skull shape in *C. taurinus*, including horns, is significantly different between sexes
2. The horns of *C. taurinus* form a distinct phenotypic module which is weakly integrated with the rest of the skull
3. The horns are positively allometric with skull size, and at a higher degree than any other skull element
4. Allometric trajectories of males and females are significantly different

Gaining a thorough understanding of the patterns of growth and shape variation of the horns of *C. taurinus*, how the horns integrate with the rest of the skull, and how secondary sexual traits differ between sexes, will resolve questions which are often overlooked or poorly understood in studies of sexually selected traits. Ultimately, the answers to these questions can be used to better understand how to detect signals of sexual selection in the morphology of both extant and extinct taxa.

## Results

**Fig. 1:**
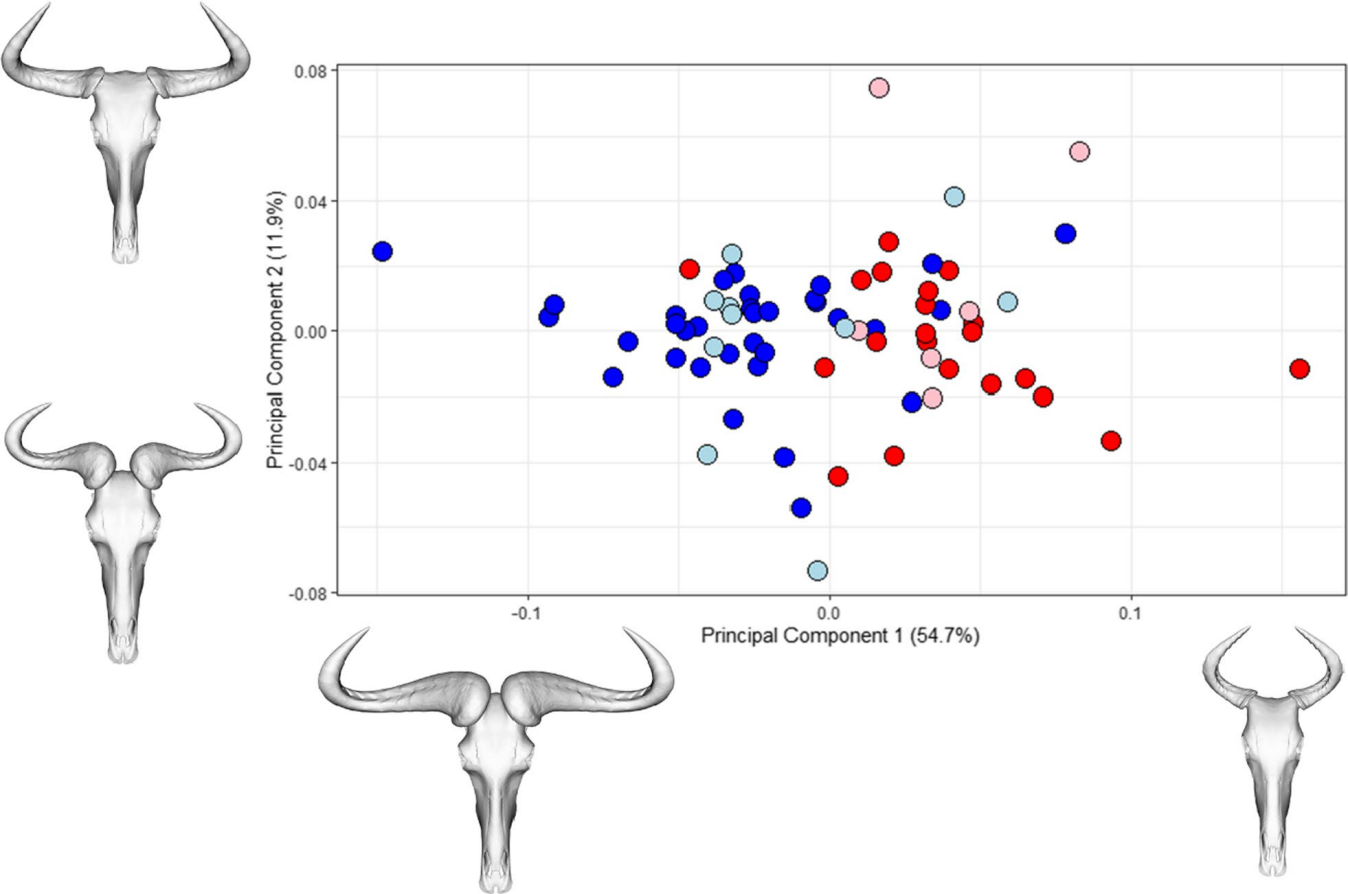
PCA of whole skull shape in *C. taurinus,* for first two principal components*.* Points are coloured according to sex (dark blue: male; light blue: probable male; red: female; pink: probable female). Projected skull shapes are shown in dorsal view along PC axes, representing extreme positive and negative shapes for each PC

Principal components analysis shows that the majority of shape variation (PC1, 57.4%) involves a change from narrow-span, small-horned shape at maximum value of PC1 to broad-span, large-horned shape at its minimum, with some corresponding relative increase in skull width around the orbits. The distribution of specimens on PC1 also suggests that sex is a discriminating factor, with female specimens (red) towards the positive end and male specimens (blue) towards the negative. PC2 (11.9% of total shape variation) shows mainly a change in horn shape, from curved horns that are flattened at the base, to more rounded horns which have a deep, pronounced boss at the contact with the skull (Fig. 1).

The MANOVA performed on the raw shape data found a significant difference in shape between male and females in all specimens (n = 70, R^2^ = 0.21, *p* = 0.001), and when specimens of uncertain sex were removed (n = 54, R^2^ = 0.24, *p* = 0.001). Similarly, male and female centroid size was found to be significantly different (n = 70, F = 42.36, *p* ≤ 0.001; Additional file 1: Fig. S4), including when analysing horns alone (F = 62.27, *p* = 0.001) and whole skull minus horns (F = 35.38, *p* = 0.001). The k-means cluster analysis performed reasonably well in identifying specimen groups, correctly assigning 81% of male and 89% of female specimens in raw shape data, and 72% of males and 93% of females in centroid size data (Additional file 1: Fig. S5). When optimum cluster number was assessed, however, results were mixed. The average silhouette method returned an optimum cluster number of 2 for the entire dataset, but also for male-only and female-only subsets of the sample (Additional file 1: Fig. S6). The Gap statistic method found no support for clustering in either the entire dataset, or male-only or female-only datasets. The dip test for non-unimodality was unable to detect non-unimodality in either shape data in the first eight PCs (Additional file 1: Table S5) or centroid size (D = 0.042, *p* = 0.48). Although strongly male-skewed, subdividing this dataset into equal numbers of each sex, or into individual sexes alone does not affect these results (Additional file 1: Tables S2–S4).

Allometry was found to have a strong and significant effect across the whole skull (n = 70, R^2^ = 0.38, *p* = 0.001). The difference in allometric slope between males and females was found to be non-significant (R^2^ = 0.01, *p* = 0.635), suggesting shared allometric trajectories. Moreover, no significant difference in shape was found between males and females when corrected for allometry (n = 70, R^2^ = 0.02, *p* = 0.206), suggesting that differences in shape between sexes is an artefact of size. This finding is further supported by the results of the k-means cluster analysis on allometry-corrected shape data, which correctly assigned only 48% of female and 56% of male specimens, values no better than random group assignment (Additional file 1: Fig. S5). The results of the optimum cluster analyses echoed the uncorrected shape data, with the average silhouette method finding a 2-cluster optimum for the entire dataset, and the gap statistic method finding no support for clusters in the allometry-corrected dataset (Additional file 1: Fig. S6).

**Fig. 2:**
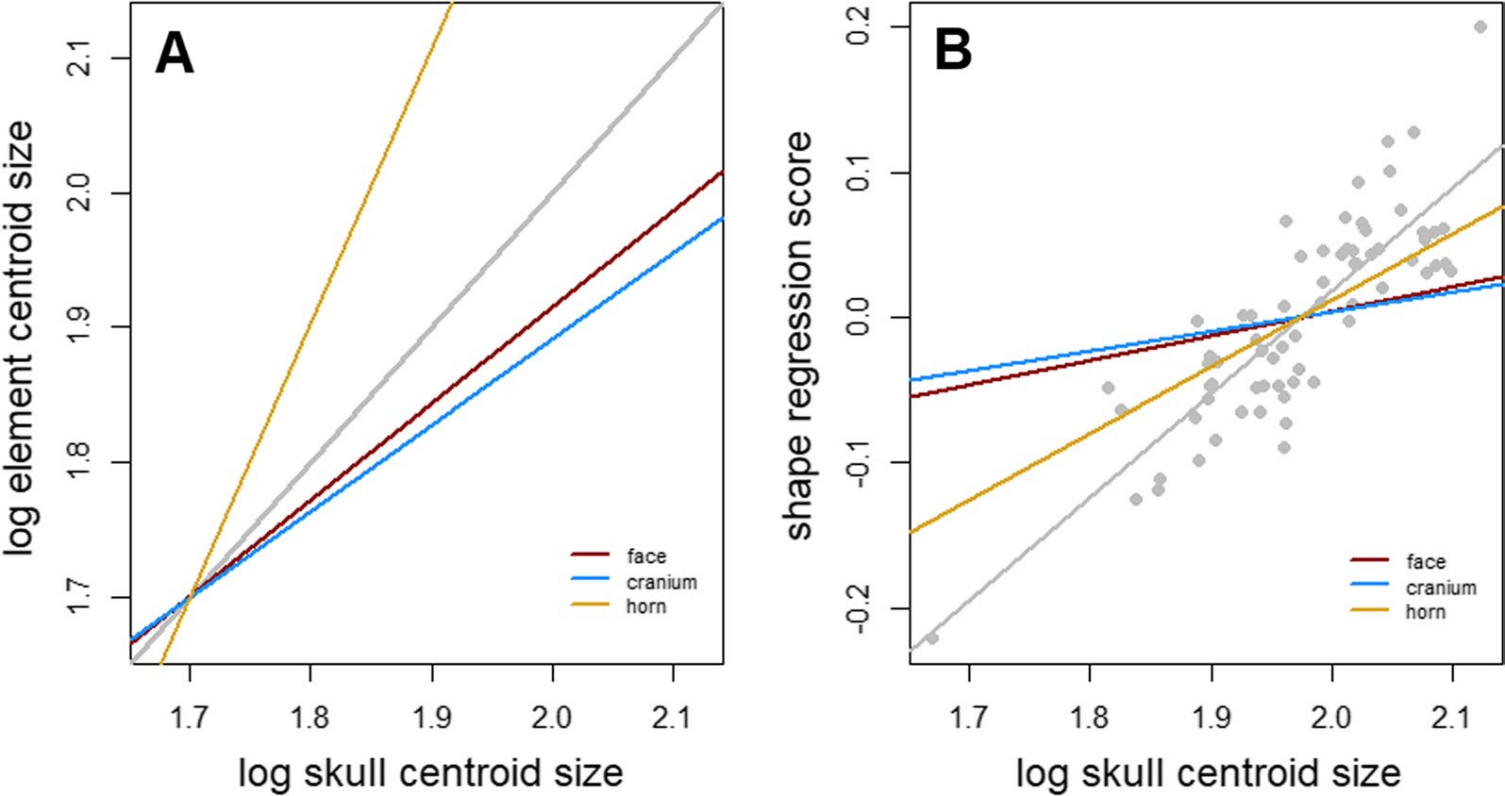
Allometry analyses of separate modules by centroid size (**A**) and shape (**B**). Slopes are coloured according to modules. Grey lines in each plot correspond to whole-skull, grey points in B represent whole-skull shape data for all specimens

**Fig. 3:**
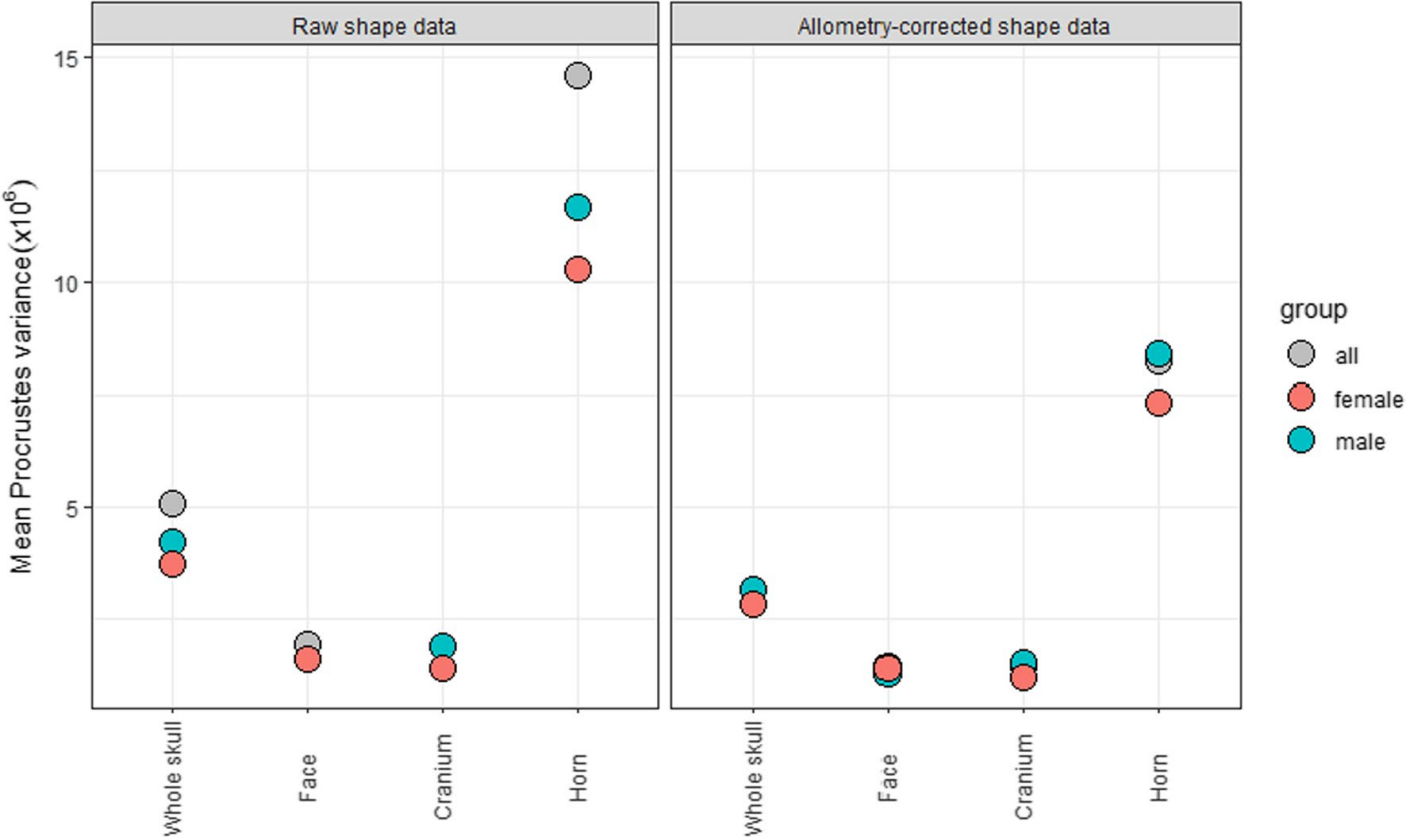
Relative per-module morphological variance. Shown are the mean Procrustes variance for globally-aligned whole skull and individual modules for raw (left plot) and allometry-corrected (right plot) shape data. Points are coloured by sex

The compare.CR analysis returned strongest support for a 3-module hypothesis (face, cranium and horns), thus supporting an integrated skull with weakly integrated horns (CR = 0.81, *p* = 0.001). The shape of all phenotypic modules were found to be significantly correlated with size, with effect size ranging from 0.22 (cranium) to 0.44 (horn; Additional file 1: Table S7). The allometric slope of the horn was significantly higher than that of any other module (*p* = 0.001), in analyses of both shape and centroid size (Fig. 2), but the difference in allometric slope between male and female horn shape was found to be non-significant (R^2^ = 0.01, *p* = 0.635). The k-means clustering analysis performed on individual modules produced mixed results in identifying sex for most modules, correctly assigning more than 75% of specimens in for horn (86%) and face (81%; Additional file 1: Table S8). As with whole-skull shape data, k-means clustering performed poorly on allometry-corrected modules, not accurately assign any sex to a greater degree of accuracy than 72% (male face, Additional file 1: table S7). Of all phenotypic modules, the horns had the highest mean shape variance (Fig. 3). The high variance of the horns remains when corrected for allometry, indicating that it is not simply a product of their greater variation due to allometry.

## Discussion

Sexual selection is predicted to have consistent and detectable effects on the morphology of secondary sexual traits, and confirming these predictions in extant taxa, for which we have detailed information, is an important step in better understanding its effects. In this study we have shown that the sexually selected horns of the blue wildebeest, *Connochaetes taurinus*, fit hypothesised patterns of growth and variation [10–12], supporting the claim that secondary sexual traits may be readily detectable using morphology alone. Of the four hypotheses outlined in the introduction, three are supported by this study. First, skull shape differs significantly between sexes in *C. taurinus*; however, this difference is not significant after correcting for allometry*.* Second, the skull of *C. taurinus* has a modular structure, with the horns forming an internally integrated module which is weakly integrated with the rest of the skull. Finally, the horns of *C. taurinus* show significant correlation with size and change shape at a higher rate than any other skull element. The fourth hypothesis, that the allometric trajectories of skull shape differ significantly between sexes is not supported, however. Our findings thus suggest that sexual dimorphism in *C. taurinus* is due to differences in size between sexes, with shape differences solely reflecting size differences due to allometry. Crucially, separating sexes within this dataset is impossible to verify without prior knowledge of sex, even with an exaggerated sexually selected trait which has been demonstrated to be sexually dimorphic. This issue is likely to apply widely unless sexual dimorphism is either in the form of presence/absence of sexually selected traits, or in extreme size dimorphism.

Intrasexual competition for mates often results in selection for larger size in the competing sex because larger individuals are physically dominant, and this may lead to the evolution of strong sexual dimorphism in size depending on the magnitude of this difference in selection [24, 37]. Weapons to aid in physical competition, such as horns, may also evolve in the competing sex, and the presence of these traits within a population is sometimes used to infer sexual selection [17, 18]. The evolution of strong positive allometry of these traits is consequently expected to evolve as a signal to amplify size and thus dominance [11, 14]. Our study supports this prediction with significant correlation of shape with size across the skull of *C. taurinus,* and the horns showing significantly higher relative rates of size and shape change than other skull elements. The increased variance of the horns compared to other skull elements, even when corrected for allometry, is another prediction of secondary sexual traits that is supported by our study. This is thought to be a result of relaxed functional constraints on the form of these traits compared with other traits [9, 11].

In some circumstances, secondary sexual traits may be expressed in the non-competing sex, but for other reasons [19, 21], and it might be expected that the degree of dimorphism in form may be amplified in such traits because of different selective pressures on each sex. When assessing sexual dimorphism in the dataset, however, our results were mixed. Although shape was found to be significantly sexually dimorphic, this was not the case when the dataset was corrected for allometry. This shift can be explained by the identical allometric trajectories of the sexes in *C. taurinus.* Similarly, the k-means clustering analysis performed well in identifying sexes in raw shape data (84% accuracy), but when shape data was corrected for allometry it performed no better than random (53% accuracy). Furthermore, the two methods employed for assessing optimum cluster number gave contradictory results that were not affected by either correcting for specimen size, or by removing either sex from the dataset entirely. These results have important implications for detecting sexual dimorphism; dimorphism can be strongly dependent on size, and allometry can act to magnify shape dimorphism between sexes.

Further complicating studies of sexual dimorphism, methods for detecting dimorphism, even in a large sample, have limitations. For example, similar to previous analyses of dimorphism, Hartigans’ dip test was particularly unsuccessful at detecting non-unimodality in this study [15]. Our dataset fits the recommended criteria outlined by Hone and Mallon [38] for detectable dimorphism, in being a large sample size (> 35 specimens) and a taxon with rapid growth to asymptotic size. Nevertheless, despite being seemingly segregated in a principal components analysis (Fig. 1), the overlap in shape and size between males and females was sufficient to mask robust recovery of dimorphism using the dip test method (Additional file 1: Table S5 and Fig. S4). Although *k*-means clustering performed well in distinguishing sexes in the raw data, the number of clusters is pre-determined by the user. When assessing sexual dimorphism, two clusters will therefore always be recovered, even when assessing a single-sex dataset, as we have demonstrated here. Furthermore, clustering accuracy can only be assessed with independent knowledge of specimen sex, which may not be available, especially for many fossil taxa [15]. In a dataset of adult specimens known to be significantly dimorphic, as in this study, this method works well in distinguishing sexes because of the difference in mean skull size between the two. In an ontogenetic study which captures the full range of morphologies from infant to fully-grown adult, it is more likely to separate juvenile and adult specimens by shape because this is often the greatest source of shape variation. *K*-means clustering analysis is therefore only appropriate where specimens are of a similar life stage, or when ontogenetic shape data are corrected for allometry.

Despite being an archetypal sexually selected structure in males, there is presently little agreement on the function of horns in female bovids [19, 20], even in well studied taxa such as *C. taurinus* [35]*.* Predator defence, male mimicry, and genetic linkage to males [35] are the most frequently cited explanations for the presence of horns in female bovids. However, predator defence is seldom observed in *C. taurinus* and is often ineffective [35], although horns may act as a visual deterrent to predators in the open habitat in which this species tends to live [19, 32]. Male mimicry in this species is predicted to allow younger males to benefit from remaining in the maternal herd for longer [35], but there are two main problems with this hypothesis. Firstly, older males apparently have little problem in distinguishing and routinely evicting older males from herds of females and young [35]. Secondly, it is unclear exactly how this would lead to the evolution of male mimicry by females rather than the evolution of female mimicry by males, given that it is the males which would benefit from remaining in a (majority) female herd. Horns in females may occur through genetic linkage, and similar expression of linked traits in both sexes is expected in this case. This is possible, but is not universal among bovids because the females of many bovid taxa are hornless [32, 33]. The presence of horns in females likely serves some adaptive function [9, 13], given the regularity with which they are lost in females of related taxa, and it is possible that female horns are maintained by a combination of factors in *C. taurinus*.

Our results reveal that the skull of *C. taurinus* has a modular structure, with skull elements forming discrete phenotypic modules which are able to grow and vary with some independence from other elements. The modularity analysis we performed supported low integration of the horn with the rest of the skull. This is likely in secondary sexual traits because this allows horns to respond to selection with some degree of independence [29]. This can explain the strong positive allometric growth of this trait, and the considerably higher morphological variance of the horns, even when corrected for allometry (Fig. 3). Modularity may ultimately allow the evolution of a wide range of horn shapes across bovidae [32, 34], and analyses of evolutionary modularity and morphological disparity across the clade will help to support this prediction. Comparing modules across the entire skull is an important step in establishing the extreme growth of sexually selected traits, because it allows us to put the sexual trait in context with other aspects of anatomy and removes the tendency to focus on a single trait, which may introduce bias into the analysis [27].

Historical collecting biases towards larger ‘trophy’ specimens [16] may have the effect of creating distinct peaks of the largest individuals of both sexes, and fewer smaller individuals, which may create an even more marked sexual dimorphism than found in a natural population by decreasing the overlap between sexes. Furthermore, the keratin horn sheath is known to vary in size across different taxa relative to the bony horn core it encloses [31], and measurements taken on the horn sheath may therefore further amplify horn allometry. It is therefore likely that in fossil or osteology specimens, where soft tissues such as keratin are not preserved, that the effects of allometry and dimorphism will be less pronounced than in the specimens used here. All analyses were performed on a global GPA of the skull and horns, and this may affect downstream results because of the redistribution of some of the high variance of the horns to other regions of the skull. This may have the effect of increasing integration across the skull, reducing variance in high-variance regions, such as the horns, and increasing in in low-variance regions [39, 40]. Despite these effects, the horns remain the most variable region of the skull, show the highest allometry, and are not strongly integrated with the rest of the skull. It is likely that separately aligning the skull elements will magnify the difference in variance and allometry between the horns and rest of the skull, but this approach will not preserve relative position and scaling of different skull elements and so is of limited use in assessing variation across the dataset [40].

## Conclusions

Sexual selection appears to have driven the evolution of both horns and sexual size dimorphism in *C. taurinus*. Our study has shown that the horns of *C. taurinus* displays patterns of growth and variation typically found in secondary sexual traits [11] in both sexes, despite strong sexual selection operating only in males. Although sexes are significantly different in size and shape, sexual dimorphism in the skull shape of *C. taurinus* appears to be a product of size, and its strong correlation with shape. Our findings show that contrary to some previous claims [17, 18], determining dimorphism is not vital in detecting the signal of sexual selection in the horns of *C. taurinus.* Both sexes follow identical patterns of growth and variation across the skull and horns and are separated only by size, suggesting linkage in trait expression between sexes in this species. Our results reflect attempts to recover dimorphism in extinct taxa where sex is not known a priori [15, 41]. These findings do not apply universally across all secondary sexual traits, and nor would they be expected to given the diversity and complexity of these traits [2, 3, 11]. Reproductive biology, life history and intensity of sexual selection may all affect the diversity, magnitude of sexual dimorphism and relative growth of secondary sexual traits, but the basic effects of sexual selection on morphology are likely to remain detectable to some extent.

## Methods

A total of 75 *C. taurinus* skulls (Additional file 1: table S1) were digitised from the collections of the Natural History Museum, London (NHM, n = 73) and the Museum Für Naturkunde, Berlin (MfN, n = 2) using photogrammetry [42]. Permission for sample collection was received from the respective museums. Meshes were decimated to one million faces and landmarks were placed on the right half of each mesh using Stratovan Checkpoint [43]. A total of 49 anatomical landmarks and 50 semilandmark curves were used to capture shape across the right side of the skull [28, 44]. Landmarks were placed on the keratinous horn sheath because it is present in all specimens and not removable. Because some important regions of the specimens (e.g. horns) do not have points or sutures suitable for placing anatomical or semilandmarks, the shape of these regions cannot be fully captured by using anatomical and semilandmark curves alone. For this reason, additional surface semilandmarks were placed on a template specimen and projected to all other specimens with the R [45] package *Morpho* [46], and following the procedure of Bardua et al. [44], giving a total of 849 fixed and semilandmarks. Missing landmarks were estimated using the thin plate spline (TPS) method in the R package *Morpho* [46] in specimens where minor damage prevented the placing of some landmarks. Several specimens (n = 5) were more severely damaged or incomplete and were thus omitted from the surface semilandmark dataset. Semilandmarks were slid to minimise bending energy. Analyses were performed on all surface semilandmarked specimens (n = 70) unless otherwise stated. All additional analyses performed on subsets of the dataset are presented in the Additional file 1: data. Landmarks were reflected across the sagittal plane and then aligned using a generalised Procrustes alignment (GPA) in the R package *geomorph* [47], and the reflected landmarks were removed, leaving the original, Procrustes-aligned right-side landmarks for analysis. This approach is common in landmark-based analyses and improves the alignment accuracy when aligning one half of a bilaterally symmetrical structure such as a skull, removes redundant shape variation introduced by including elements from both sides, and allows the inclusion of specimens which are damaged or incomplete on one side [41, 44]. Asymmetry is not accounted for by this approach, but inspection of specimens revealed a lack of any obvious asymmetry, and did not lead us to suspect that this will have a significant effect on our results. We performed a global GPA to assess shape variation across the entire skull, and downstream analyses were performed on this globally-aligned data, including those performed on subsets of the shape data. Global Procrustes alignment retains relative positional and scaling information across a structure, maintaining covariance values between different regions [39, 40]. A global GPA may increase overall integration across a structure by redistributing high regional variance, and separately aligning different regions is therefore likely to relax integration between these regions, and may limit analyses that are designed to investigate morphological variation across regions.

All further analyses were performed using R Statistical Software [45]. Figures were produced in *ggplot2* [48].

A principal components analysis (PCA) was performed on the Procrustes-aligned dataset to determine major trends in shape variation. Sexual shape dimorphism was assessed with a Procrustes multivariate analysis of variance (MANOVA) with the ‘procD.lm’ function in *geomorph* [47], using known sex of each specimen as the independent grouping factor. Firstly, a k-means cluster analysis [49] was performed on the Procrustes-aligned shape data, with the *k* value (i.e. number of expected groups) set at 2, representing two expected sexes. The k-means cluster analysis was repeated on centroid size (defined as the sum of the squared distance of each landmark to the geometric centre of each specimen, [50]) for all specimens, with *k* value again set at 2. To assess whether number of groups could be obtained from the data without prior knowledge of group number, the optimum number of clusters was assessed using a k-means approach with two commonly-used methods, the average silhouette method [51] and the gap statistic method [52]. The entire dataset and subsets of the data containing exclusively male and female specimens were analysed with these approaches to determine that two-group and no-group datasets could be distinguished. Finally, Hartigans’ dip test was performed on the first 8 principal components of the Procrustes-aligned shape data to test for non-unimodality with the R package *diptest* [53]. To test for non-unimodality in specimen size, the dip test was also performed on the centroid sizes of all specimens.

Phenotypic modularity was assessed by comparing four modularity hypotheses, comprised of subsets of the landmark data, with the compare.CR function in *geomorph* [47]. Hypotheses were designed to test integration across the skull and horns by comparing a fully-modular hypothesis (all bones separate modules) with three more integrated combinations of the skull elements with horns variably integrated with other skull elements ([29]; Additional file 1). The globally-aligned modules identified in this analysis were subjected to a k-means cluster analysis to assess grouping by sex for each module [49].

Allometry in the dataset was explored by regressing shape with size, using the function procd.lm in the R package *geomorph* [47]. Differences in allometric slope between sexes was assessed by including sex as a factor in the regression. Allometry analyses were repeated for each globally-aligned module defined in the modularity analysis. Additionally, centroid sizes of each module were regressed against whole-skull centroid size to assess size-based allometry, comparable to the more traditional approach employed by O’Brien et al. [11]. Shape data were corrected for allometry by using the residuals obtained from the allometry regression to produce a size-independent shape dataset [54]. Sexual dimorphism was assessed in the allometry-corrected dataset by repeating the dimorphism analyses performed on the raw shape data. Specifically, a Procrustes MANOVA was performed using sex as the independent grouping factor, a k-means cluster analysis was performed with a *k* value of 2 with results compared to observed specimen sex, and optimum cluster number was assessed using the average silhouette [51] and gap statistic [52] methods. Finally, Hartigans’ dip test was used to assess non-unimodality in the allometry-corrected shape dataset for the first 8 residual shape components, and in the centroid size data [53].

Finally, to compare relative levels of shape variance across the skull we used the ‘morphol.disparity’ function in *geomorph* [47], for whole-skull and individual module shape data derived from the modularity analysis. Values were divided by the number of landmarks in each module to give a mean variance value that could be compared across different modules, and was repeated for allometry-corrected shape data [40].

## Supplementary Information

**Additional file 1:** Supporting methods data and additional results.

## Data Availability

Scan data used in this study will be available for download from www.phenome10k.org, subject to copyright rules of the respective repositories.

## Abbreviations

CR: Covariance ratio
GPA: Generalised Procrustes alignment
MANOVA: Multivariate analysis of variance
MfN/ZMB: Museum für Naturkunde, Berlin
ML: Maximum likelihood
NHM: Natural History Museum, London
PCA/PC: Principal component analysis/Principal component
