# Supplementary material for: Sexual selection leads to positive allometry but not sexual dimorphism in the expression of horn shape in the blue wildebeest, Connochaetes taurinus
Source: BMC Ecol Evol. 2022 Sep 11;22:107. doi: 10.1186/s12862-022-02060-3 (PMC9464394; doi:10.1186/s12862-022-02060-3)
Supplement: Supplementary file 1 — Additional file 1: Supporting methods data and additional results. [file 12862_2022_2060_MOESM1_ESM.docx]

**Sexual selection leads to positive allometry but not sexual dimorphism in the expression of horn shape in the blue wildebeest, *Connochaetes taurinus.***

**Chloé Gerstenhaber and Andrew Knapp**

**Supplementary data**

**Note on sample and additional analyses**

The sample consists of 75 *C. taurinus* skulls; 47 males and 28 females. Of these specimens, 5 were too severely damaged to place surface semilandmarks and so were excluded from the surface semilandmark analysis (i.e. **patched specimens**), and were analysed only with anatomical landmarks and semilandmark curves (i.e. **curves only**). Sex was known for certain (i.e., information was included on specimen labels) for 36 males and 22 female specimens. The sex of the remaining specimens was estimated by the authors and marked as ‘probable male’ or ‘probable female.’ The dataset was subdivided to include only specimens of certain sex (i.e. ‘**strict**’ as opposed to ‘**all**,’ which also included specimens with estimated sex), and analyses were rerun on the ‘**strict**’ subset. In addition, all analyses were run on individual sexes (**Males only** and **Females only**), for all specimens and only those of known sex. Because males outnumber females, the largest males were removed from the dataset to equalise the number of each sex (‘**equal sample**’), for both the ‘**all**’ and ‘**strict**’ subsets.

Specimens in the dataset represent all 5 subspecies of *C. taurinus*: *C. t. taurinus* (n = 36)*, C. t. johnstoni* (n = 4)*, C. t. mearnsi* (n = 4)*, C. t. albojubatus* (n = 6) and *C. t. cooksoni* (n = 3)*.* In addition, 15 specimens are of unknown subspecies. To account for possible differences in shape between these subspecies a MANOVA was applied to the entire dataset with subspecies as the independent grouping variable, which did not reveal a significant difference between any group (Supplementary Data Table S3). Nevertheless, all analyses were repeated solely on specimens of the best-represented subspecies, *C. t. taurinus* (n = 36). These analyses are reported as ‘**subspecies analysis**’ in the results tables below. The results of these analyses did not differ from our analysis of the entire dataset. Subspecies of *C. taurinus* are mainly distinguished by colouration and median body weight (Castelló, 2016), with horn shape being somewhat distinct in the Western White wildebeest, *C. t. mearnsi* (Estes, 2014; Castelló, 2016). Differences in horn shape that are independent of size are seen along PC2 of Fig. 1 of the main text, but are not sufficient to distinguish subspecies in this dataset. It may be the case that increasing the sample sizes of different subspecies may reveal more reliable differences between groups in this way.

**Table S1: Specimen data.** F = female, M = male, F_p = probable female, M_p = probable male.

**Anatomical landmark guide**

1. Anterior tip of nasal on midline
2. Anterior lateral process of nasal
3. Posterior angle of nasal at contact with frontal
4. Posterior point of nasal on midline
5. Posterior dorsal angle of premaxilla at contact with maxilla
6. Anterior dorsal angle of premaxilla
7. Anterior tip of premaxilla on midline
8. Posterior ventral angle of premaxilla at lower lateral contact with maxilla
9. Dorsal anterior corner of premaxilla at nasal/premaxilla contact
10. Dorsal posterior corner of maxilla at contact with lachrymal
11. Posterior-most contact of maxilla with zygomatic at underside of zygomatic arch
12. Posterior-most point of tooth row on maxilla
13. Anterior lateral contact of maxilla with premaxilla on ventral curve
14. Zygomatic contact with frontal on orbit margin
15. Posterior-most point of zygomatic contact with temporal on ventral part of zygomatic arch
16. Junction between zygomatic/maxilla/lachrymal on zygomatic
17. Junction of zygomatic and lachrymal on anterior orbit margin
18. Junction of lachrymal and zygomatic on anterior orbit margin
19. Junction of lachrymal and frontal on orbit margin
20. Anterior-most angle of dorsal edge of lachrymal
21. Ventral anterior angle of lachrymal at junction with zygomatic and maxilla
22. Anterior point of frontal on dorsal midline of skull
23. Posterior point of frontal on dorsal midline of skull
24. Anterior junction of frontal and temporal on inside margin of orbit
25. Posterior contact between frontal and zygomatic on posterior vertical bar of orbit
26. Anterior lateral-most point of frontal at junction of lachrymal and nasal
27. Anterior midline point of parietal at contact with frontal
28. Posterior midline point of parietal at contact with occipital
29. Lateral point of parietal at contact with temporal
30. Anterior-most point of parietal at contact with frontal
31. Anterior point at base of horn sheath
32. Posterior point at base of horn sheath
33. Tip of horn
34. Midline contact of occipital with parietal
35. Midline point of occipital at edge of foramen magnum
36. Tip of occipital condyle on edge of foramen magnum
37. Midline of occipital at ventral contact with sphenoid
38. Tip of paraoccipital process
39. Posterior contact point of temporal with parietal
40. Anterior contact point of temporal with parietal
41. Midline ventral contact of sphenoid with occipital
42. Ventral contact of sphenoid with palatine on edge of lateral flange
43. Midline posterior point of palatine
44. Midline anterior point of palatine
45. Midline posterior point of maxilla on ventral surface of skull
46. Ventral anterior contact of maxilla with premaxilla on underside of skull
47. Posterior-most point of ventral premaxilla process on underside of skull
48. Anterior point of midline premaxilla contact on underside of skull
49. Anterior-most point of premaxilla on underside of skull

**Semilandmark curve guide (see anatomical landmarks guide for identification of points)**

1. Points 1 to 2 along anterior edge of nasal
2. Points 2 to 3 along lateral edge of nasal
3. Points 3 to 4 along posterior edge of nasal
4. Points 4 to 1 along medial edge of nasal
5. Points 5 to 6 along dorsal edge of premaxilla
6. Points 6 to 7 along dorsal edge of premaxilla
7. Points 7 to 8 along ventral edge of premaxilla
8. Points 8 to 5 along posterior edge of premaxilla
9. Points 9 to 10 along dorsal edge of maxilla
10. Points 10 to 11 along posterior suture of maxilla
11. Points 11 to 12 along posterior limit of maxilla
12. Points 12 to 13 along lateral edge of tooth row
13. Points 13 to 9 along anterior suture of maxilla
14. Points 14 to 15 along rear margin of vertical orbit bar and suture with zygomatic process of temporal
15. Points 15 to 16 along ventral suture of zygomatic
16. Points 16 to 17 along zygomatic suture with lachrymal
17. Points 17 to 14 along orbit margin
18. Points 18 to 19 along orbit margin
19. Points 19 to 20 along dorsal lachrymal suture with frontal
20. Points 20 to 21 along anterior lachrymal suture with maxilla
21. Points 21 to 18 along ventral lachrymal suture with zygomatic
22. Points 22 to 23 along midline frontal suture
23. Points 23 to 24 along frontal/parietal suture
24. Points 24 to 25 along posterior margin of posterior vertical orbit bar
25. Points 25 to 26 along orbit margin and frontal/lachrymal suture
26. Points 26 to 22 along frontal/nasal suture
27. Points 27 to 28 along dorsal midline of parietal
28. Points 29 to 30 along parietal/occipital suture
29. Points 30 to 31 along parietal/temporal suture
30. Points 30 to 27 along parietal/frontal suture
31. Points 31 to 32 around posterior base of horn sheath
32. Points 32 to 31 around anterior base of horn sheath
33. Points 31 to 33 along dorsal surface of horn
34. Points 33 to 32 along ventral surface of horn
35. Points 34 to 35 along dorsal midline of occipital
36. Points 35 to 36 along border of foramen magnum
37. Points 36 to 37 along foramen magnum and ventral midline of occipital
38. Points 37 to 38 along occipital/sphenoid suture
39. Points 38 to 34 along anterior suture of occipital
40. Points 39 to 40 along temporal/parietal suture
41. Points 40 to 39 along zygomatic process of temporal and temporal occipital suture
42. Points 41 to 42 along suture of sphenoid and palatine
43. Points 42 to 41 along edge of lateral flange of sphenoid
44. Points 43 to 44 along toothrow and palatine/maxilla suture on underside of skull
45. Points 44 to 43 along midline palatine suture
46. Points 45 to 46 along ventral midline maxilla suture
47. Points 46 to 45 along toothrow and maxilla/palatine suture on underside of skull
48. Points 47 to 48 along midline premaxilla suture on underside of skull
49. Points 48 to 49 along medial edge of premaxilla
50. Points 49 to 47 along ventral suture between premaxilla and maxilla

**Modularity tests**

**Skull elements**

1: Nasal

2: Premaxilla

3: Maxilla

4: Zygomatic

5: Lacrimal

6: Frontal

7: Parietal

8: Horn

9: Occipital

10: Temporal

11: Sphenoid

12: Palatine

**Modularity hypotheses tested with compare covariance ratio method (compare.CR).** Numbers in parenthesis correspond to skull elements listed above.

1. All bones separate modules (1:12); **12 modules**
2. **1.** Horn (8), **2.** all other bones (1:7, 9:12); **2 modules** (integrated skull, horns as separate module)
3. **1.** Face (1:5,12), **2.** cranium (6,7,9:11), **3.** horn (8); **3 modules** (skull split into two modules, anterior and posterior, with horns as separate module)
4. **1.** Face (1:5,12), **2.** horn + cranium (6:11); **2 modules** (skull split into two modules, anterior and posterior, with horns integrated with cranium)

**Table S2: Results of allometry analyses on shape data.**

|  | **Curves only** | | | **Patched specimens** | | |
| --- | --- | --- | --- | --- | --- | --- |
|  | **n** | **R^2^** | ***p*** | **n** | **R^2^** | ***p*** |
| **M+F - All** | 75 | 0.22 | 0.001 | 70 | 0.38 | 0.001 |
| **M+F – All, equal sample** | 56 | 0.18 | 0.001 | 54 | 0.33 | 0.001 |
| **M+F - Strict** | 58 | 0.22 | 0.001 | 54 | 0.37 | 0.001 |
| **M+F – Strict, equal sample** | 44 | 0.20 | 0.001 | 42 | 0.36 | 0.001 |
| **Males only – All** | 47 | 0.14 | 0.001 | 43 | 0.25 | 0.001 |
| **Males only - Strict** | 36 | 0.14 | 0.001 | 33 | 0.25 | 0.001 |
| **Females only – All** | 28 | 0.15 | 0.001 | 27 | 0.24 | 0.001 |
| **Females only - Strict** | 22 | 0.15 | 0.007 | 21 | 0.31 | 0.002 |
| **Subspecies analysis (*C. t. taurinus* only)** | n/a | n/a | n/a | 36 | 0.37 | 0.001 |

**Table S3: Comparison of Allometric slopes by sex.**

|  | **Curves only** | | | **Patched specimens** | | |
| --- | --- | --- | --- | --- | --- | --- |
|  | **n** | **R^2^** | ***p*** | **n** | **R^2^** | ***p*** |
| **All** | 75 | 0.01 | 0.366 | 70 | 0.01 | 0.635 |
| **All, equal samples** | 56 | 0.03 | 0.017 | 54 | 0.02 | 0.145 |
| **All, horns only** | n/a | n/a | n/a | 70 | 0.003 | 0.848 |
| **Strict** | 58 | 0.01 | 0.464 | 54 | 0.01 | 0.605 |
| **Strict, equal samples** | 44 | 0.03 | 0.046 | 42 | 0.02 | 0.116 |
| **Subspecies analysis** | n/a | n/a | n/a | 36 | 0.03 | 0.146 |

**Table S4: MANOVA analyses of differences in shape by sex and subspecies.** Significant results are shown in **bold.**

|  | **Curves only** | | | **Patched specimens** | | |
| --- | --- | --- | --- | --- | --- | --- |
|  | **n** | **R^2^** | ***p*** | **n** | **R^2^** | ***p*** |
| **All** | 75 | 0.16 | **0.001** | 70 | 0.21 | **0.001** |
| **All, equal samples** | 56 | 0.12 | **0.001** | 54 | 0.15 | **0.001** |
| **All, allometry corrected** | 75 | 0.04 | **0.005** | 70 | 0.02 | 0.206 |
| **All, equal samples, allometry corrected** | 56 | 0.06 | **0.001** | 54 | 0.04 | 0.052 |
| **Strict** | 58 | 0.19 | **0.001** | 54 | 0.24 | **0.001** |
| **Strict, equal samples** | 44 | 0.17 | **0.001** | 42 | 0.19 | **0.001** |
| **Strict, allometry corrected** | 58 | 0.05 | **0.005** | 54 | 0.03 | 0.139 |
| **Strict, equal samples, allometry corrected** | 44 | 0.07 | **0.004** | 42 | 0.04 | 0.126 |
| **Subspecies analysis, all specimens** | n/a | n/a | n/a | 53 | 0.11 | 0.107 |
| ***C. t. taurinus* subset, sex** | n/a | n/a | n/a | 36 | 0.25 | **0.001** |


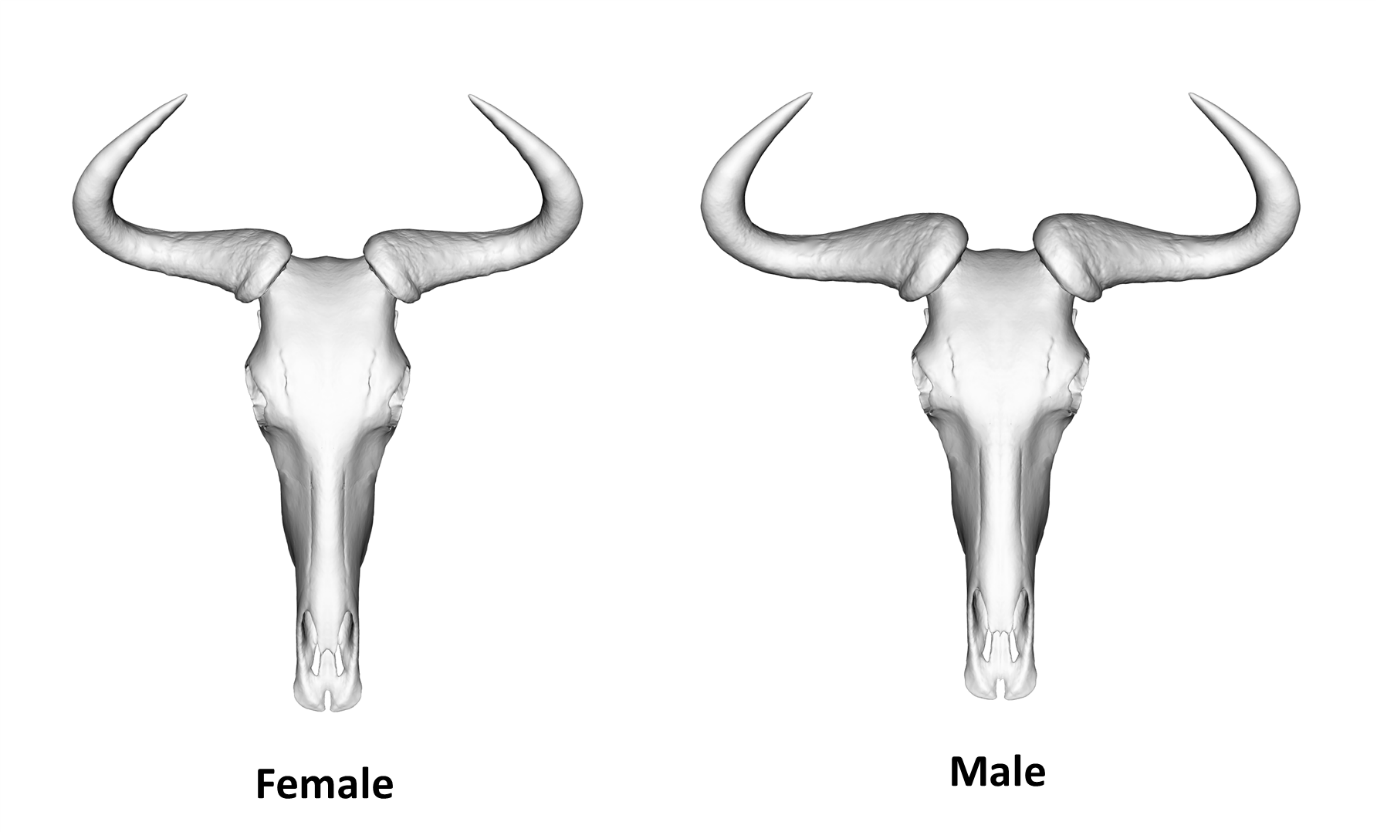


**Figure S1: Warped meshes representing mean shape of female (left) and male (right) *C. taurinus* skull.**


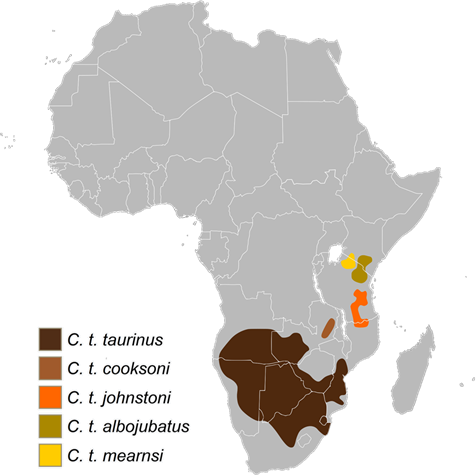


**Figure S2: Distribution of *C. taurinus* subspecies across Africa (image licensed under CC BY-SA 3.0).**


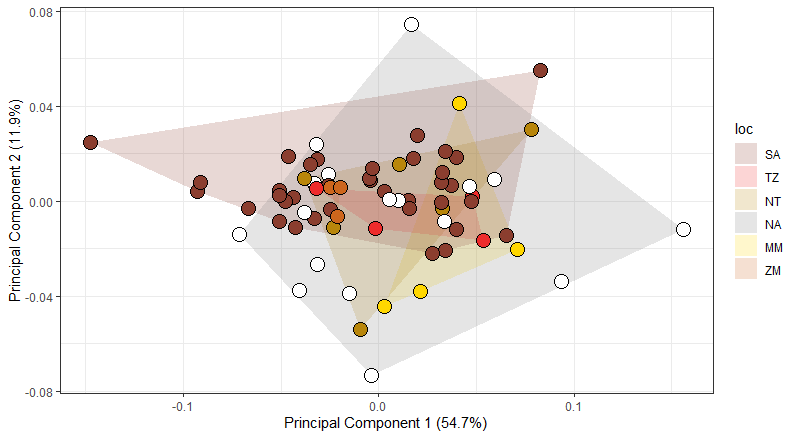


**Figure S3: PCA of specimens coloured by subspecies.** Convex hulls group subspecies, SA: *C. t. taurinus ;* TZ: *C. t. johnstoni*; NT: *C. t. albojubatus*; MM: *C. t. mearnsi*; ZM: *C. t. cooksoni;* NA: not known.

**Table S5: Results of dip tests performed on shape and size data.**

|  | **Raw shape data** | | | **Allometry-corrected data** | | |
| --- | --- | --- | --- | --- | --- | --- |
|  | **PC proportion of variance (%)** | **D** | ***p*** | **PC proportion of variance (%)** | **D** | ***p*** |
| **Principal component 1** | 54.7 | 0.046 | 0.34 | 31.9 | 0.041 | 0.55 |
| **Principal component 2** | 11.9 | 0.029 | 0.98 | 18.4 | 0.044 | 0.38 |
| **Principal component 3** | 7.3 | 0.042 | 0.51 | 11.1 | 0.030 | 0.95 |
| **Principal component 4** | 4.3 | 0.039 | 0.65 | 6.0 | 0.024 | 1.00 |
| **Principal component 5** | 3.1 | 0.035 | 0.80 | 4.3 | 0.036 | 0.76 |
| **Principal component 6** | 2.6 | 0.029 | 0.98 | 4.1 | 0.034 | 0.86 |
| **Principal component 7** | 1.6 | 0.036 | 0.80 | 2.6 | 0.049 | 0.23 |
| **Principal component 8** | 1.5 | 0.034 | 0.83 | 2.1 | 0.032 | 0.89 |
| **Dip test, All, centroid size** | n/a | 0.042 | 0.48 | n/a | n/a | n/a |


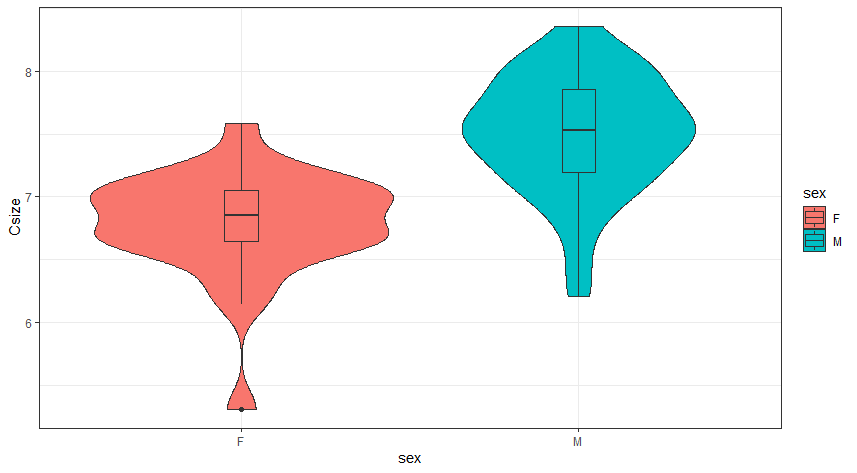


**Figure S4: Violin plots of centroid size distributions for females (F) and males (M).**

**Table S6: ANOVA of centroid size by sex**

|  | **Curves only** | | **Patched specimens** | |
| --- | --- | --- | --- | --- |
|  | **F** | ***p*** | **F** | ***p*** |
| **All** | 34.2 | <0.001 | 42.36 | <0.001 |
| **Strict** | 30.72 | <0.001 | 40.55 | <0.001 |
| **All, horns only** | n/a | n/a | 62.27 | <0.001 |
| **All, skull minus horns** | n/a | n/a | 35.38 | <0.001 |

**Table S7: Results of shape allometry analysis of individual modules for all specimens, derived from compare.CR analysis.**

|  | ***n*** | **R^2^** | ***p*** |
| --- | --- | --- | --- |
| **Face** | 70 | 0.27 | 0.004 |
| **Cranium** | 70 | 0.22 | 0.004 |
| **Horn** | 70 | 0.44 | 0.004 |

**Table S8: Results of k-means cluster analysis on whole-skull and individual module shape data, for raw and allometry-corrected shape data.** Results show percentage of individuals correctly assigned to group. Values above 75% are shown in bold.

|  | **Raw shape data** | | | **Allometry corrected shape data** | | |
| --- | --- | --- | --- | --- | --- | --- |
|  | **Males % correct** | **Females % correct** | **Total % correct** | **Males % correct** | **Females % correct** | **Total % correct** |
| **Whole skull** | **81%** | **89%** | **84%** | 56% | 48% | 53% |
| **Horn** | **79%** | **96%** | **86%** | 56% | 48% | 53% |
| **Face** | **81%** | **81%** | **81%** | 72% | 67% | 70% |
| **Cranium** | 74% | 74% | 74% | 47% | 67% | 59% |
| **Centroid size, whole skull** | 72% | **93%** | **80%** | n/a | | |

**
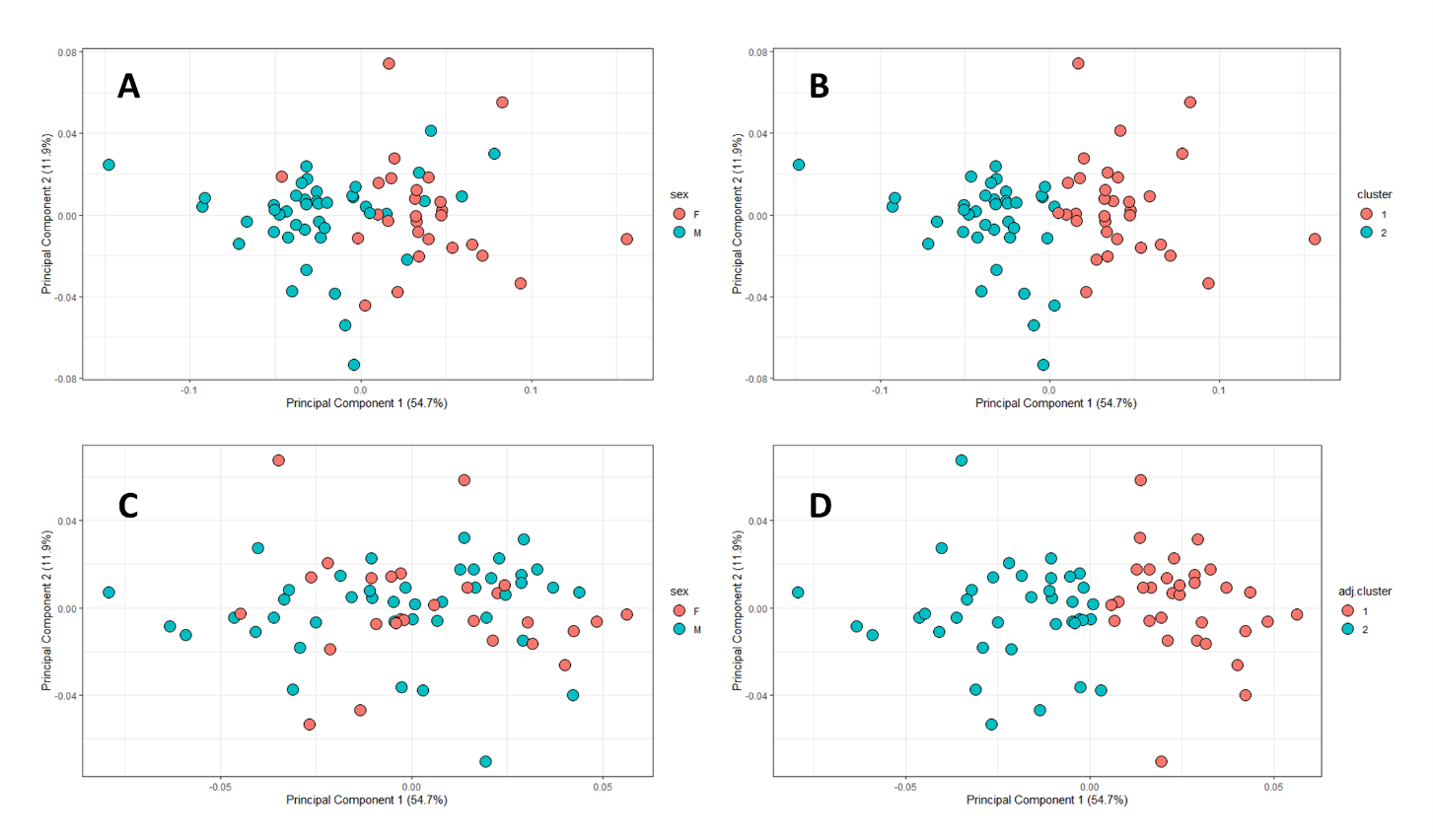
**

**Figure S5: Results of *K*-means cluster analysis of sex for n = 2 clusters, whole—skull shape data.** Shown are PCAs of **A:** Raw shape data, coloured by known sex; **B:** Raw shape data, coloured by k-means cluster analysis estimate of sex; **C:** Allometry-corrected shape data, coloured by known sex; **D:** Allometry-corrected shape data, coloured by k-means cluster analysis estimate of sex.

**
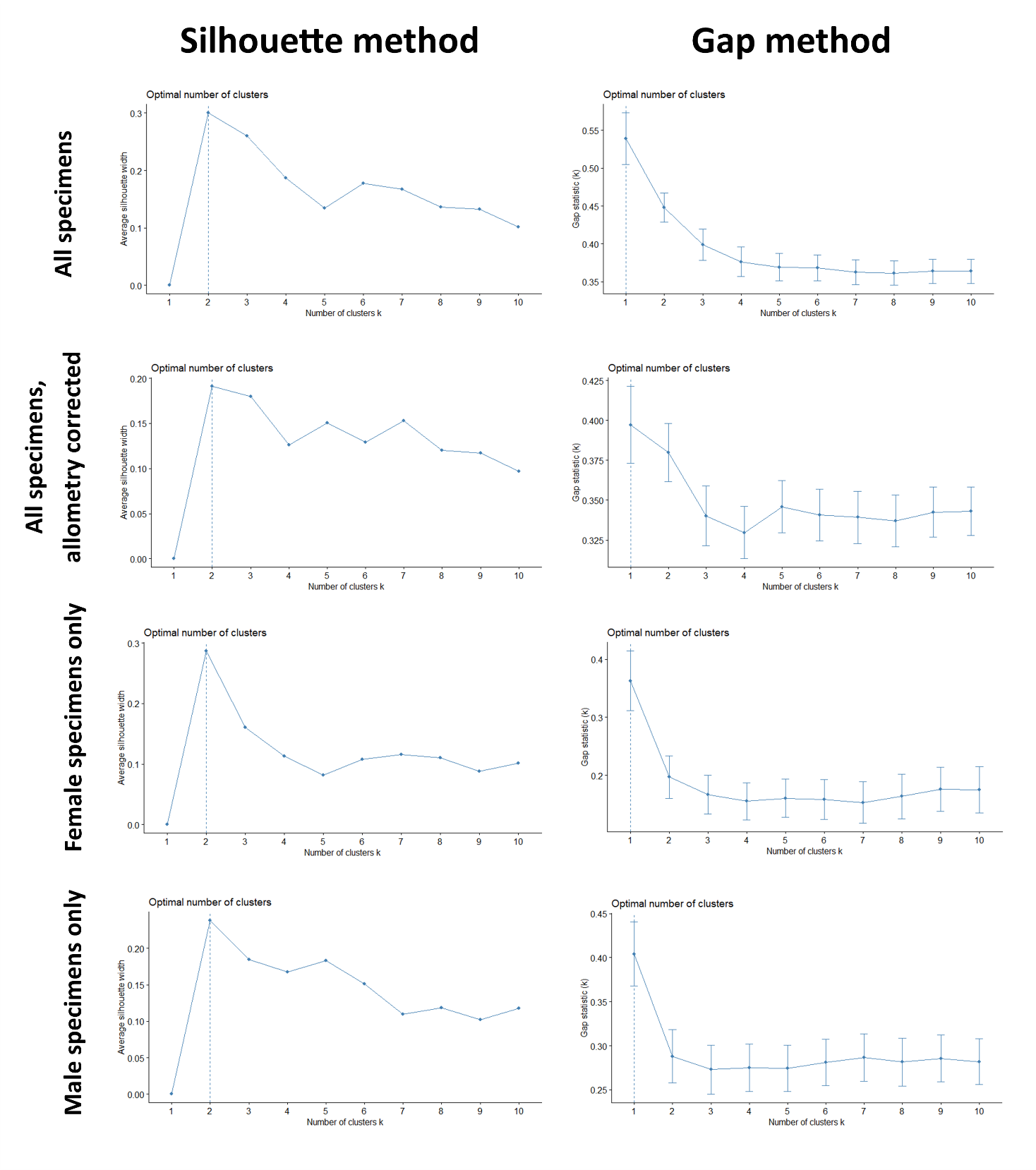
**

**Figure S6: Results of optimum cluster number analysis.** Left column shows results of ‘silhouette’ method for (top to bottom) all specimens (n = 70), allometry corrected specimens (n = 70), female specimens only (n = 27) male specimens only (n = 43). Right column shows results for ‘gap’ method for same subdivisions of dataset. Vertical dashed line in each plot represents calculated optimum cluster number.

**Table S9: Morphological variance of whole skull and individual modules, for all data and separate sexes.**

|  | **Raw data** | | | **Allometry corrected** | | |
| --- | --- | --- | --- | --- | --- | --- |
|  | **All** | **Males** | **Females** | **All** | **Males** | **Females** |
| **Whole skull** | 5.10 | 4.22 | 3.72 | 3.15 | 3.15 | 2.82 |
| **Face** | 1.95 | 1.62 | 1.60 | 1.43 | 1.28 | 1.42 |
| **Cranium** | 1.88 | 1.89 | 1.42 | 1.46 | 1.53 | 1.19 |
| **Horn** | 14.61 | 11.66 | 10.29 | 8.24 | 8.40 | 7.33 |
